# Cerebral Aspergillosis Caused by *Neosartorya hiratsukae*, Brazil

**DOI:** 10.3201/eid0809.020073

**Published:** 2002-09

**Authors:** Josep Guarro, Esper G. Kallas, Patricio Godoy, Anna Karenina, Josepa Gené, Alberto Stchigel, Arnaldo Lopes Colombo

**Affiliations:** * Unitat Rovira i Virgili, Reus, Spain; † Escola Paulista de Medicina-UNIFESP, São Paulo, Brazil; ‡ Hospital do Servidor Público Etatual de São Paulo, Brazil

**Keywords:** Aspergillus, mycoses, cerebral infection, antifungal agents

## Abstract

We report the first case of infection by *Neosartorya hiratsukae*, an ascomycete in which the conidial state resembles *Aspergillus fumigatus*. The fungus caused a brain infection in a Brazilian woman, who died despite itraconazole treatment. Diagnosis was established by direct microscopic examination, computed tomographic scan, and magnetic resonance imaging of the brain, and repeated cultures from the lesions. The in vitro antifungal susceptibility of the isolate is provided.

*Aspergillus fumigatus* is the most common filamentous fungus to cause opportunistic infections in humans. Two close relatives of *A. fumigatus*, classified in the ascomycetous genus *Neosartorya*, have been documented to cause occasional opportunistic infections (1). These species are *N. fischeri* and *N. pseudofischeri*. The former has been reported on two occasions as causing systemic infection in transplant recipients (2, 3), as well as a mixed pulmonary infection in a patient with myeloma (4). *N. pseudofischeri* has been reported to cause different localized and invasive infections (5–9). The conidial states of these species are morphologically very similar to that of *A. fumigatus.*

We describe the first cerebral infection caused by another species of *Neosartorya*, *N. hiratsukae*. This taxon has been described only in Japan, where it was isolated from air and from pasteurized aloe juice (10).

## Case Report

A 75-year-old Brazilian woman was admitted to the Hospital do Servidor Público Estadual de São Paulo on May 5, 1999, with progressive memory loss, confusion, and involuntary movements in the upper right arm after a fall 1 year earlier. She had also developed gait disorder, with short steps and constant loss of balance that led to a diagnosis of Parkinson’s disease in a neurology consultation in March 1999; however, the condition did not respond to the usual treatment. On April 30, a computed tomographic (CT) scan of the brain showed multiple lesions in both brain hemispheres, after which the patient was referred to the hospital. Past clinical history showed an evaluation of productive cough in 1996, with bloody sputum, night sweats, and intermittent fever; she underwent bronchoscopy with pathologic examination, which showed vascular congestion and focal intra-alveolar edema, but no specific pathogen was identified.

**Figure 1:**
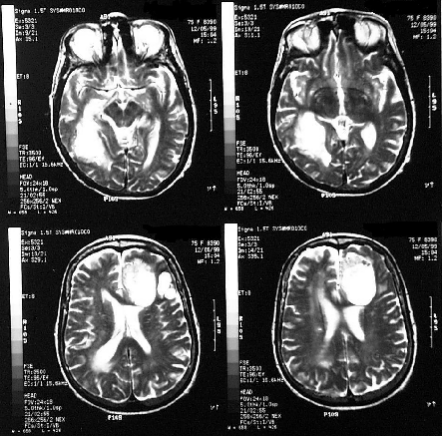
Magnetic resonance imaging (MRI) of the brain obtained after first drainage.

On examination, the patient appeared chronically ill, mildly pale, disoriented, and confused, although she was able to follow simple commands. The lungs had decreased sounds in both lower thirds, with rales. The upper arms moved slowly and repetitively, with a loss of strength. Tendon reflexes were normal. The patient underwent surgical exploration, with drainage of the frontal and occipital lesions. Four samples of a yellowish, dense liquid were collected. Laboratory examination did not show neoplastic cells or neutrophils in the liquid. Direct microscopic examination showed septate hyphae in all the samples. Cultures were negative for aerobic and anaerobic bacteria and mycobacteria, and two samples were positive for a fungus, tentatively identified as *Aspergillus* sp. The patient was initially treated for 21 days with ceftriaxone, oxacillin, and metronidazole without major improvement. When the fungus was isolated, she was treated with amphotericin B and underwent postdrainage magnetic resonance imaging (MRI) of the brain, which showed multifocal brain abscesses, including subtentorial and supratentorial lesions (Figure 1). A chest x-ray and a CT scan of the thorax showed a small, bilateral pleural effusion and a left pulmonary cavitary lesion. Left pleural drainage obtained 80 mL of a clear yellow pleural effusion with pH 7.5, 19,870 leukocytes/mL and 970 erythrocytes/mL, with negative cultures for mycobacteria, aerobic and anaerobic bacteria, and fungi. Refractory hypokalemia developed after the patient received a total dose of 1 g of amphotericin B, which led to discontinuation of this drug. A course of amphotericin B plus itraconazole (400 mg/day) was initiated.

On the first day of itraconazole therapy, another MRI of the brain was performed, which showed an increase in the volume of the abscesses. During the next few days, the patient’s mental state worsened, and she had another cerebral drainage 8 days later. A yellowish opaque liquid, with a white granular deposit, was obtained from the frontal and parietal abscesses. A cerebral fragment was obtained for analysis, which showed only reactional brain tissue. All cultures were negative for bacteria but positive for the same fungus that had been isolated previously. Her clinical and neurologic status improved, and she was discharged to a nursing home on July 20, 1999; itraconazole (400 mg/day) treatment was continued.

The patient was again admitted to the hospital on August 8, and she stayed for 21 days after being diagnosed with urinary sepsis caused by *Escherichia coli*. She was successfully treated with intravenous ceftriaxone. Her neurologic condition had been stable since her previous hospital discharge. She was last seen at the outpatient clinic on September 29, with an unchanged neurologic condition. During the hospital stay, another MRI of the brain showed a decrease in the size of all lesions, which was interpreted as a good response to the itraconazole therapy. Because of the difficulties of maintaining home care, she was admitted to another nursing hospital. Her clinical state deteriorated, and she died in November 1999 from multiple organ failure. Clinical information on this last hospital admission is very limited, and a postmortem examination was not performed.

Cultures obtained on the two occasions yielded molds with identical morphologic features, and one isolate was referred to the Medical School of Rovira i Virgili University in Reus, Tarragona, Spain, for identification purposes.

The isolate was subcultured on Czapek agar and malt extract agar (MEA), and incubated at approximately 25°C in the dark. After 14 days, the colonies on Czapek agar were very restricted (12 mm–14 mm in diameter), velvety, irregularly folded, umbonate, and white to yellowish white, with a pale yellow reverse. Sporulation was absent. On MEA, the colonies developed rapidly, attaining a diameter of 40 mm–46 mm in 14 days. They were velvety, radially folded, white to greenish white, with a pale yellow reverse. Ascomata and conidial heads developed throughout the culture. The fungus grew restrictedly at 45°C.

The microscopic features of ascomata and conidial heads were examined from wet mounts prepared in lactic acid under a light microscopy. Ascomata were non-ostiolate, superficial, white to light cream colored, globose or subglobose, measuring 120 µm–600 µm in diameter, and covered with a white aerial mycelium. The peridium was thin and membranous. The asci were eight-spored, more or less globose, and measured 11µm–15 µm in diameter. The ascospores were hyaline, one-celled, and lenticular, with two closely pressed equatorial crests. They measured 6 µm–7.5 µm x 4 µm–5 µm, including the crests, and their convex walls showed a fine reticulate ornamentation. Numerous conidiophores of an *Aspergillus* sp. were intermixed with the ascomata. The conidiophores consisted of green to bluish green, uniseriate, short columnar conidial heads over hyaline to light green, smooth and thick-walled stipes, which measured 100 µm–170 µm x 3 µm–4 µm. The conidia were pale greenish, globose or subglobose, 2 µm–2.5 µm in diameter and with smooth or delicately roughened walls.

**Figure 2:**
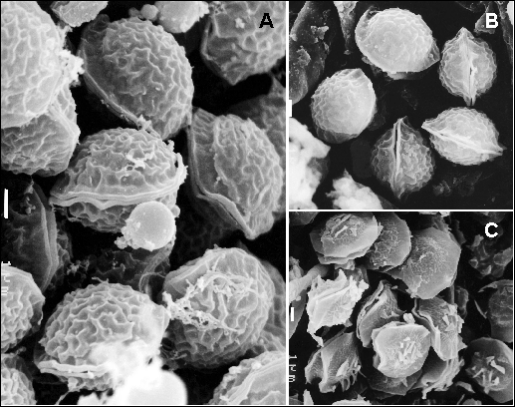
Ascospores of *Neosartorya hiratsukae*, CBS 109356 (A) and NHL 3008 (B), and of *N. pseudofischeri*, NRRL 3496 (C), under scanning electron microscopy. Bars A, B, C = 1 µm.

On the basis of the above characteristics, and especially taking into account the ascospore ornamentation observed under scanning electron microscopy (Figure 2), we identified the isolate as *N. hiratsukae* (10,11). The isolate was morphologically compared with the type strain of *N. hiratsukae* (NHL 3008) and was proven to be the same species (Figure 2A,B). In addition, *N. hiratsukae* is the only species of *Neosartorya* with reticulated ascospores that grow restrictedly on Czapek agar, a characteristic also shown in the case isolate. *N. pseudofischeri,* the most common *Neosartorya* species involved in human infections, is easily distinguished because its ascospore walls are ornamented with raised flaps of tissue resembling triangular projections or long ridge lines (Figure 2C). Living cultures of the case strain are deposited in the Centraalbureau voor Schimmelcultures, the Netherlands (CBS 109356) and in the Institute of Hygiene and Epidemiology, Belgium (IHEM 18438).

The case isolate was tested to determine its susceptibility to five antifungal drugs. Tests were carried out by a microdilution method described previously (12) and adapted from the reference method for molds recommended by the National Committee for Clinical Laboratory Standards (13), with RPMI 1640 medium buffered to pH 7.0 with 0.165 M morpholinepropanesulfonic acid, an inoculum of 9.1 x 10^5^ CFU/mL, an incubation temperature of 30°C, a second-day reading (48 h), and an additive drug-dilution procedure. MICs and minimum fungicidal concentrations (MFC) were as follows: amphotericin B 1 and >16 μg/mL, flucytosine 64 and >64 µg/mL, itraconazole 0.25 and 0.25 µg/mL, voriconazole 0.25 and 0.5 µg/mL, and UR-9825 0.06 and 0.5 µg/mL, respectively. Results demonstrated good activity of the four-azole derivatives tested. UR-9825, a novel triazole not yet licensed, showed the lowest MIC. MFCs of amphotericin B and flucytosine were very high, indicating the ineffectiveness of these drugs. These data correlate with our clinical results since a total dose of 1 g of amphotericin B was unable to reduce the brain abscesses, while the patient responded well to itraconazole at daily doses of 400 mg.

This case report is important because such clinical isolates of *Neosartorya* spp. that produce white colonies and do not become green, like colonies of *A. fumigatus*, are often discarded as contaminants. Therefore, the real incidence of aspergillosis caused by *Neosartorya* species could be underreported. Many *Neosartorya* species, other than *N. fischeri* or *N*. *pseudofischeri*, are thermotolerant and can grow at temperatures above 37°C, showing their inherent ability to invade the brain.
